# PyParadigm—A Python Library to Build Screens in a Declarative Way

**DOI:** 10.3389/fninf.2019.00059

**Published:** 2019-08-09

**Authors:** Felix G. Knorr, Johannes Petzold, Michael Marxen

**Affiliations:** Department of Psychiatry and Neuroimaging Center, Technische Universität Dresden, Dresden, Germany

**Keywords:** Python, library, experimental psychology, paradigm, declarative UI, 2D

## Abstract

In experimental psychology, subjects are often confronted with computer-based experimental paradigms. Creating such paradigms can require a lot of effort. PyParadigm is a newly developed Python library to ease the development of such paradigms by employing a declarative approach to build user interfaces (UIs). Paradigm specifications in this approach requires much less code and training than in alternative libraries. Although PyParadigm was initially developed for the creation of experimental paradigms, it is generally suited to build UIs that display or interact with 2D objects.

## 1. Introduction

In experimental psychology, subjects are confronted with computer-based experimental paradigms. A paradigm usually consists of multiple different states in which stimuli are displayed, and the user's task is to react to the stimuli by using a response device, usually a keyboard or a pad with a few buttons. It can be thought of as something between a mini game and an interactive Powerpoint presentation. Most stimuli are either (colored) text, images, or geometric primitives, sometimes sounds are used as stimuli too. The primary goal of a paradigm is to quantify the users behavior in form of reaction times or made decisions, for example. The results need to be stored; a popular format would be CSV or TSV files.

Creating such paradigms can require quite an effort and a number of software tools have been developed to this end. Two very popular commercial ones are E-Prime and Presentation. Both of them consist of a graphical user interface (GUI), which, in case of E-Prime, even allows to specify a paradigm by dragging and dropping visual elements into a sequence, and a run-time environment, which can execute previously defined paradigms, and must be present on the PC that executes the paradigm. However, in many cases, knowledge of an integrated scripting language is required. But if it is necessary to program anyway, using a fully fledged general purpose programming language has obvious advantages. There are many programming languages that could be used, but one of the favorites in the field is Python. Besides being free, it has a reputation of being simple to learn and easy to read. Additionally it is a popular tool for data analysis, and a large number of high quality, freely available libraries exist. Thus, it is a useful addition to a scientist's skill set.

There are many popular libraries for python to create GUIs, the two most prominent ones probably being Qt and TKinter. A GUI normally runs in potentially multiple windows and consists of predefined elements like text fields and buttons, which are clicked using the mouse. Often deeply nested elements are required and elaborate methods are needed for a variety of graphical input options. An experimental paradigm, in contrast, runs in full-screen mode and requires often simpler and different types of graphical elements or input options. In addition, the temporal sequence of events is of primary importance.

Consequently, a number of libraries specifically for creation of paradigms have been developed : PsychoPy (Peirce, 2009), Expyriment (Krause and Lindemann, 2014), and VisionEgg (Straw, 2008). For a comparison (see Table 1). These libraries all work in a very similar way: Stimuli are loaded and then positioned on the screen using absolute or relative x and y coordinates. All of these libraries have a particular strength: PsychoPy has a GUI builder that will generate Python code, Expyriment can run on Android, and puts extra effort in synchronizing display time stamps with the screen refresh rate, and VisionEgg puts an emphasis on using the GPU and supports 3D graphics.

**Table 1:** Comparison of the features the different paradigm programming libraries offer.

	**PsychoPy**	**VisionEgg**	**Expyriment**	**PyParadigm**
Positioning with (x, y) coordinates	✓	✓	✓	✓^*^
Positioning via layouts	✗	✗	✗	✓
GPU acceleration	✓	✓	✓	✓^*^
3D support	✓	✓	✗	✗
GUI builder	✓	✗	✗	✗
Android support	✗	✗	✓	✗
v-sync timing synchronization	✓	✗	✓	✗
Support for blocks, run, and trials	✓	✗	✓	✗
Support for rendering numpy arrays	✓	✗	✗	✓
Support for text input	✗	✗	✓	✓
Direct interactivity with pygame	✗	✗	✗	✓

A ^*^ means: yes, through pygame.

While all of these libraries have their own advantages, they are relatively complex, need a fair amount of training to master, and require more lines of code than needed in many cases. We developed PyParadigm with the goal of being able to write paradigms with a minimum of code and training. Less code means faster development, and less room for bugs. PyParadigm can also increase readability, once familiar with it (it takes ~ 1 h to go through our tutorial at: https://pyparadigm.readthedocs.io/en/latest/tutorial.html). The main idea in PyParadigm is that images shown as stimuli are specified declaratively—not by issuing commands to draw specific parts of images but by specifying their nested structure. PyParadigm is a very thin wrapper around the video game development library pygame and uses its Surface class to represent visual data. This means that it can freely interact with pygame directly where PyParadigm falls short. For example, freely moving visual stimuli cannot be programmed using the layouting mechanisms of PyParadigm. But they can be easily implemented using pygame and integrated into PyParadigm. Additionally, PyParadigm also accepts 2D-numpy arrays as image data, which makes it easy to write scripts that utilize image data, e.g., browsing 2D-slices of a 3D MRI volume using a moving marker.

## 2. The Library

In this section, we will present PyParadigm in depth. It is published on PyPi and can be installed via pip (Python 3 only): *pip install pyparadigm*. PyParadigm is split into four modules: (1) *surface_composition*, (2) *eventlistener*, (3) *misc*, and (4) *extras*.

The surface_composition module is used to create images that are subsequently displayed on the screen. The eventlistener module handles keyboard input. The misc module contains functions to create the window, draw images within the window and a few others. The extras module contains code that has extra dependencies besides pygame, which are not installed automatically (currently numpy and matplotlib). The full documentation can be found at https://pyparadigm.readthedocs.io. In the following, you will be introduced to the important principles of PyParadigm, based on concrete examples which should enable you to write paradigms yourself with only little additional reference to the documentation or the tutorial.

### 2.1. The Surface_Composition Module

This module is the heart of PyParadigm. The idea is to describe an image with a hierarchical tree structure. Every element in the tree is assigned a part of the image to draw on, additionally it can assign any part of its space to its child elements, if it has any. The elements of which a screen is composed are not actual visual data that is stored in memory and copied onto the image, they are rendering instructions.

There are a few important subgroups of elements: layouts, wrapper, and primitives. Layouts have the sole purpose of dividing their space among their children, and enable the creation of resolution independent visuals. There are 3 different layout types: LinLayout arranges its children in a line, that is either horizontal or vertical. By default the available space is split equally among the children. The LLItem class can be used to change the proportions of the children or insert empty cells, GridLayout arranges its children on a grid, proportions can be defined for rows and columns, Overlay draws its children on top of each other.

Wrappers take a single child item (which can also be a layout) and modify the rendering. For example, they pad the element with a buffer space (Padding), reduce the available area to the biggest rectangle with defined side proportions (RectangleShaper), surround the area with a border (Border) or fill the background with a given color (Fill). One very special wrapper is the Surface, which will automatically wrap a pygame.Surface, i.e., an image that was loaded from disc or created otherwise. Images have a resolution, and cannot easily be scaled, because this might lead to bad image quality if the available space is bigger than the image, or distortions. Therefore, the default behavior is to scale images down, to the biggest possible rectangle that can be fit into the available space without distorting the image, if the available space is smaller than the image. If the available space is larger than the image it will be centered but not upscaled. The detailed behavior of how an image should be inserted can be specified through the various parameters of Surface, if it is used to wrap a pygame.Surface manually.

Primitives do not accept any children and simply render something into the assigned area, using as much space as possible. Available primitives are Circle, Cross, Line, Text, and Rectangles (by using Fill).

### 2.2. The Eventlistener Module

Input processing works in the following way: if a key is pressed, this information goes to the operating system, where it is processed. Unless it is a control sequence, like *Win + L*, the command will be sent to the window that has input focus (in this case: the pygame display window). These events are gathered in an event-queue, which is controlled by pygame. To react to events they need to be polled from the queue, then their information needs to be parsed and, consequently, actions can be taken. This process needs to be repeated periodically. Pygame provides abstractions for this by means of the EventListener class. An EventListener object will offer multiple methods. Each of them will parse the event-queue repeatedly until the event-of-interest occurs. In this case, they will return the information that was contained in the event. Additionally a timeout can be specified, which will cause the method to return if no event-of-interest occurred during the provided timeout. Available methods are for example, wait_for_keys(), which will return when one of multiple provided keys was pressed once, wait_for_unicode_char(), which will return if the user presses any key, that represents a Unicode char. This is the method of choice to get text-input from the user since it returns the unicode character instead of a pygame key-code. For mouse input, a wrapper element for the render tree is provided, which will give all its space to its child, and calls a callback function, if a mouse event that corresponds to its area occurs.

In case functionality is required that is not provided by the currently available methods, a handler function can be defined, and passed as argument to the listen() method. These functions will then be confronted with each event and can react to it in a customized way. The EventListener can take a list of handler functions on instantiation, which will always be used when the event loop is parsed.

### 2.3. The Misc Module

This module contains a collection of functions that are useful, but do not fit well into one of the other modules. It contains functions to create the display window, draw a pygame.Surface onto the screen, create a pygame.Surface of the size of the display window that is filled with a specified color, manipulate a string buffer according to a provided Unicode character and display multiple frames in a list successively.

### 2.4. The Extras Module

The *extras* module contains functions to use *numpy* arrays as images and may be extended by the user as needed. This module can be used, for example, to work interactively with data represented as numpy arrays, like browsing neuroimaging data, to extract information from volumes of choice. It could also be used to render stimuli that cannot be easily created with the surface_composition module because they based on a complex mathematical formula.

## 3. Creating a Paradigm

To demonstrate that it is very simple to create a paradigm with *PyParadigm*, we will walk the reader through the basic elements of a paradigm in this section. First we create the display window calling init(), and an EventListener instance. The rest of the main function below looks like a table of contents:


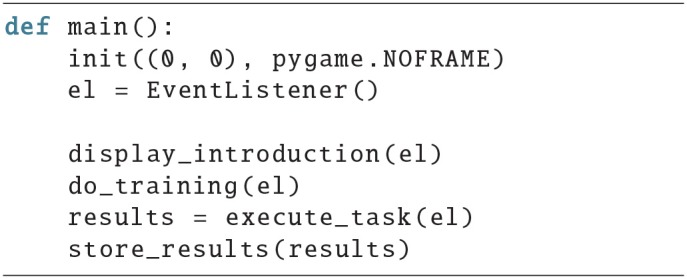


An introduction is displayed; there is a training period followed by the task; lastly, the behavioral results are saved. The introduction could look something like this:


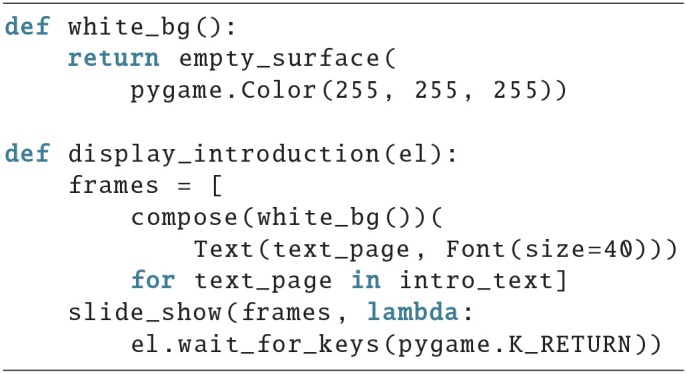


First, the frames with the text that should be displayed are created, then slide_show() is used to display all of them. A lambda function is provided that will return once the subject presses the return key, to get to the next text page. intro_text is a list of strings, where every string contains the text for one frame.

The execute_task function could look like this:


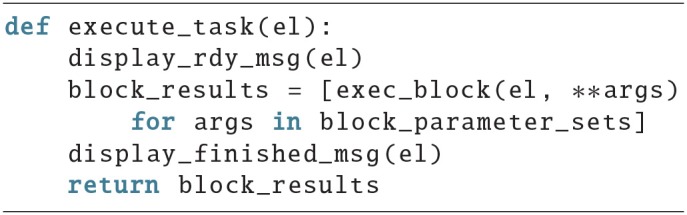


It displays a text to prepare the subject that the task will start, calls the function that will execute a single block in a list comprehension with the parameters for each block, which might be defined as a global variable in the beginning of a script, and in the end displays another message.

Here is an example for the exec_block() function:


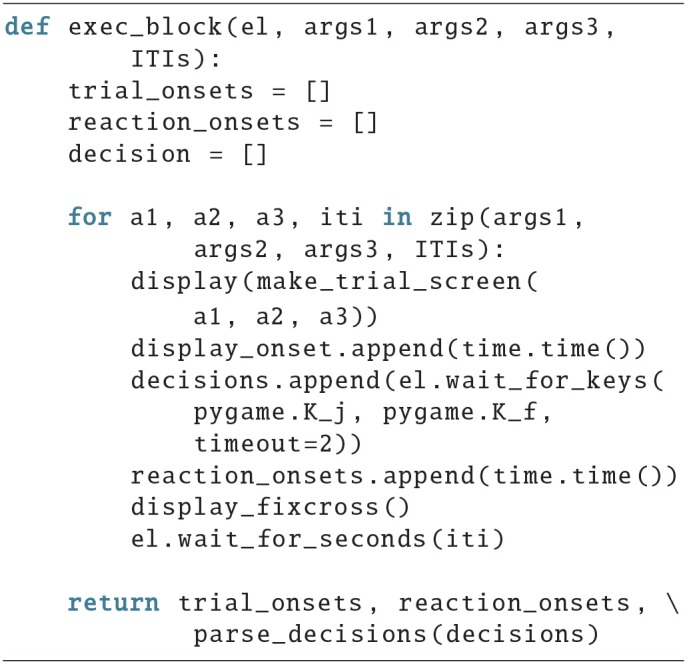


It manages the execution of a block, i.e., it calls a function to display a trial, stores an onset, waits for a response, stores the reaction time, and in the end returns the results. ITIs stands for inter trial interval and is a list of seconds which represents the duration that the subject has to wait for between two trials. The *n*th entry represents the waiting time after the *n*th trial, therefore the last value in the list should be zero.

To be able to run this code, the user still has to provide a number of variables, like a subject id, which could be passed as an argument to the script, or be queried via the input() function. It also misses the code to actually render a trial (make_trial_screen(p1, p2, p3)). Possible examples for the make_trial_screen(p1, p2, p3) function will be provided in the next section.

A fully functioning example for a real world paradigm can be found at https://github.com/KnorrFG/set_switch_paradigm/tree/master/felix_2. The implementation took roughly 1 week.

## 4. Examples

In this section, we provide some source code together with images of the resulting displays to illustrate the declarative nature and code efficiency of the library.

### 4.1. Example 1

In the first example, we create a screen for an inter-temporal choice task, in which subjects have to make a decision between receiving a small of money immediately or a larger amount after some waiting time.


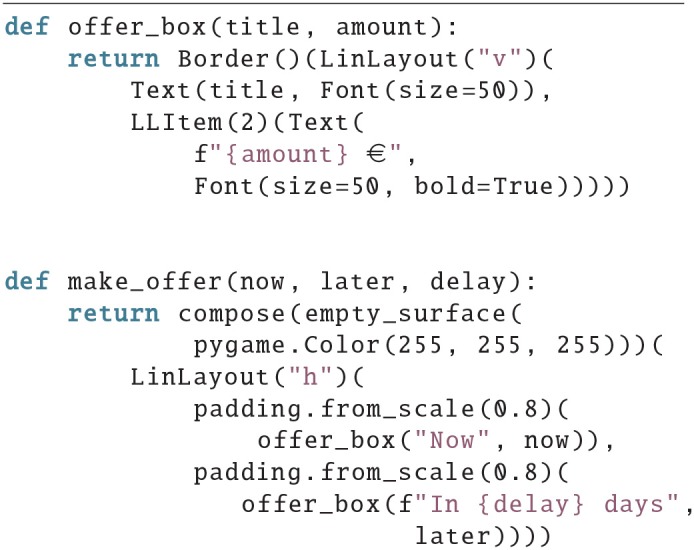


**Figure 1:**
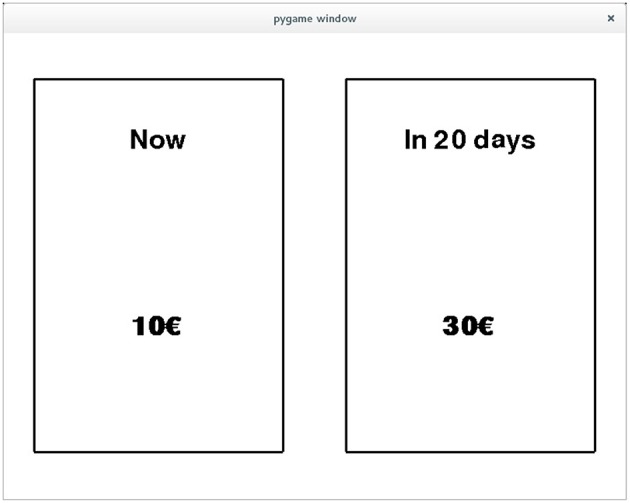
An exemplary screen of an inter temporal choice paradigm, where the subject is tasked to decide between 10€ that are payed out immediately and 20€ that are payed out after 30 days.

In the make_offer() function the screen is first divided horizontally into two parts, then the space for both is scaled down to 80% and filled with an offer box, which is a box (the Border() around the LinLayout) that contains two Text items, both of font size 50, and the amount has a bold type face. Also the space for the amount is twice as large as the space for the delay, which is implied by LLitem(2) and leads to the positioning seen in Figure 1.

### 4.2. Example 2

**Figure 2:**
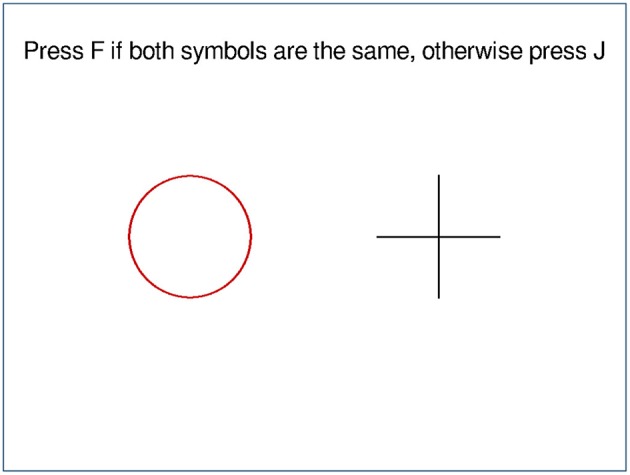
Screen of example 2.

In this example, two symbols are displayed, and the subjects have to press either the F-key or the J-key, depending on whether the symbols are identical. The output can be seen in Figure 2 and the source code below. First, the screen is divided vertically (LinLayout(“v”)), then the instruction text is added, implicitly with a proportion of one, then the two symbols are added. They are wrapped in a horizontal LinLayout, which also contains three empty LLItems to create the visible spacing. In the end, another empty LLItem is added to the outer layout, to shift everything upwards. This time, the color for the background image is provided as a hex-code, which is shorter than using pygame.Color(), also the outer LinLayout is now moved to the second argument of compose, which saves one indention level.


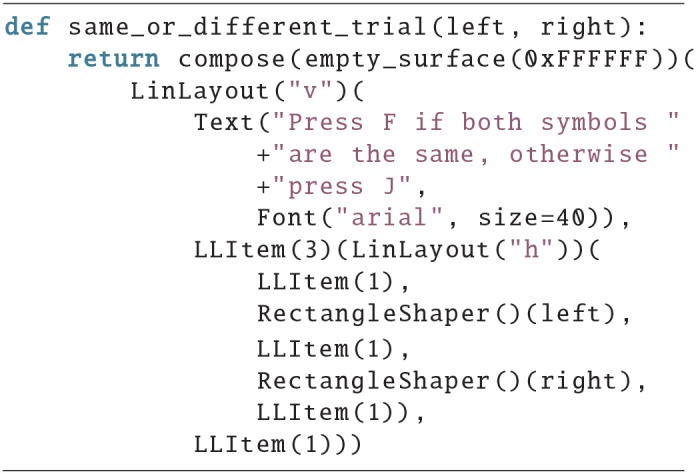


### 4.3. Example 3

**Figure 3:**
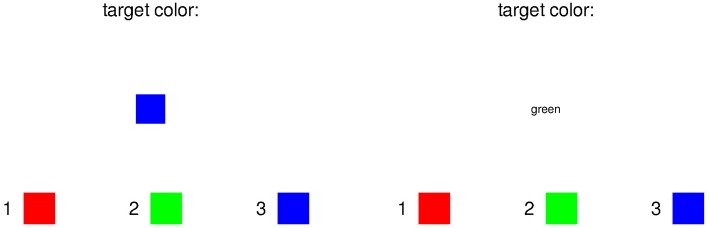
Screen of example 3.

Example 3 shows the training stage of a Stroop task, where subjects have to learn the color mappings by heart. The output is shown in Figure 3.


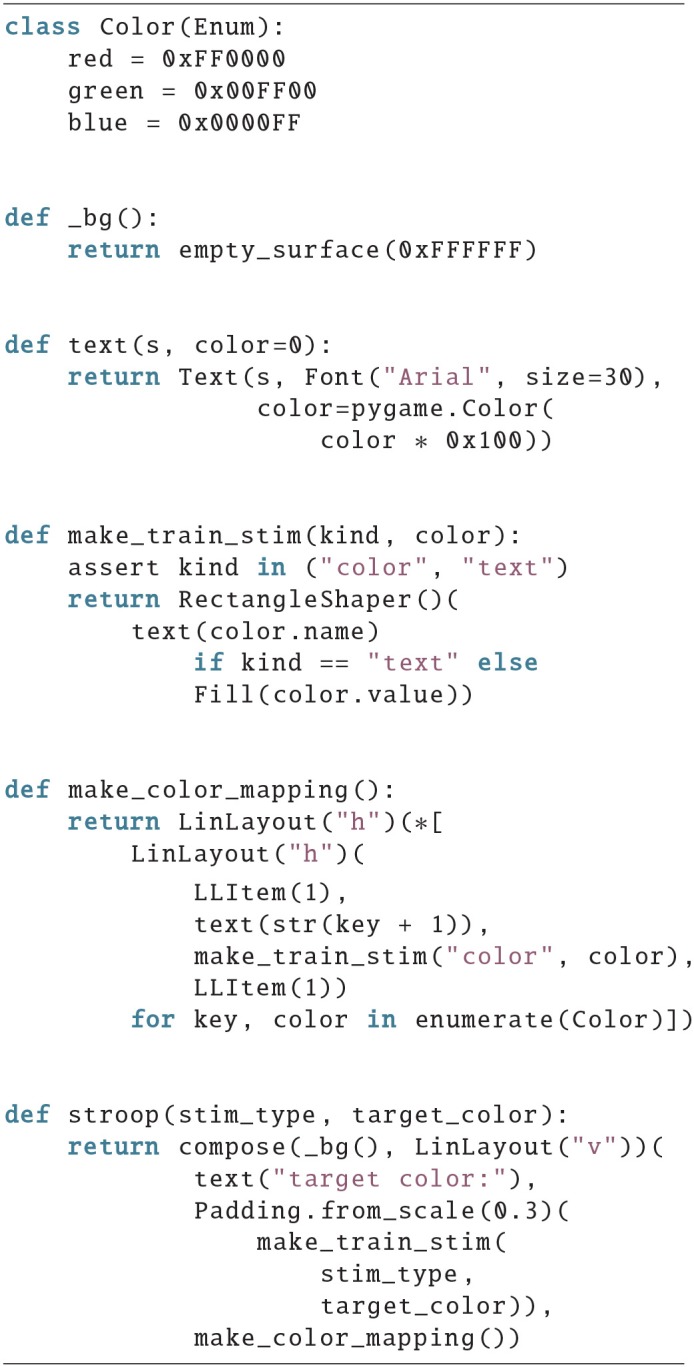


Here, two screens are provided in the output, the left one is created by the call stroop(“color”, Color.green) and the right one by stroop(“text”, Color.green). The basic layout is just 3 vertically aligned items of equal size. Then the text “target color:” is added. Next a box with the given color, or its name as text, is defined in the function make_train_stim(). At last the mapping, displaying which key to press for which color, is created in the bottom by make_color_mapping(). The mapping is created as a horizontal layout that has one horizontal layout as child for each possible color. The inner horizontal layout uses two empty LLItems on the outsides as buffers, and has a text, and a colored square in the middle. This leads to a distribution of one empty LLItem on the outsides, but two empty LLItems between 2 mappings.

## 5. Limitations

While PyParadigm is capable to create most psychological standard tests as it is, it still has some limitations. PyParadigm currently only supports keyboard and mouse input. So any external input device that does not create key-press events would require the creation of additional handler functions, probably using pygames joystick module.

Another limitation is that we did not perform any measurements of input or display delays for two reasons: 1. these delays are highly dependent on the executing hardware, 2. compared to the variance in human behavior the variance in hardware delays is low and does usually not have a negative impact on statistical outcomes (Damian, 2010). We rely on pygame's default plotting mechanisms, which usually means an instantaneous screen update when display() is called, but a delay of the screen update when the pygame.DOUBLEBUF is used for window creation. While this is no problem for most paradigms, it matters in studies using subliminal priming. Therefore, we cannot recommend using PyParadigm for such studies.

Additionally, some people may expect some classes or functions to handle the structure of a paradigm, like runs and blogs that may or may not use different conditions. We found that there is no real advantage in using something like this opposed to simply using for-loops and itertools.product, which is part of the python standard library. However, if you prefer to use such functions, we recommend to use the library “experimentator” (Harrison, 2018), which was explicitly built for this purpose. It is completely independent from PyParadigm, and since there are no overlapping functionalities, it should work together without any problems. However, we haven't tested this.

pygame does not support the serial port, but there is a library, PySerial Liechti (2019), that can be utilized to this end.

Although PyParadigm is based on pygame, which is tested thoroughly, the underlying technology is complex, and depends on the operating system, the hardware and the vendors drivers. Therefore, in some rare cases, PyParadigm might not function on some systems.

## 6. Conclusion

In this paper, we have presented our newly developed library, PyParadigm, an efficient, minimalistic and easy to learn approach to paradigm implementation. We demonstrated that the process of creating a paradigm is very straight forward. We introduced the principles of the different modules, gave a breakdown of how paradigm creation works in general, and provided three concrete examples for the surface_composition module. A real-world paradigm, which was used in a study, can be found at https://github.com/KnorrFG/set_switch_paradigm/tree/master/felix_2 to browse the source. PyParadigm is designed to save developers time in creating paradigms and to minimize the required lines of code by using an efficient composition mechanism for 2D UIs. Critical feedback via https://github.com/KnorrFG/pyparadigm and extensions of the package are very welcome.

## Author Contributions

FK designed and implemented the library, and wrote the manuscript. JP tested all provided examples and ensured that all examples are understandable to a layperson as well as proofread and improved the manuscript. MM contributed to the design of the software and provided improvements to the manuscript.

### Conflict of Interest Statement

The authors declare that the research was conducted in the absence of any commercial or financial relationships that could be construed as a potential conflict of interest.

## References

[B1] Damian M. F. (2010). Does variability in human performance outweigh imprecision in response devices such as computer keyboards? Behav. Res. Methods 42, 205–211. 10.3758/BRM.42.1.205 20160300

[B2] Harrison H. S. (2018). Experimentator. Available online at: https://github.com/hsharrison/experimentator

[B3] Krause F. Lindemann O. (2014). Expyriment: a Python library for cognitive and neuroscientific experiments. Behav. Res. Methods 46, 416–428. 10.3758/s13428-013-0390-6 24142834

[B4] Liechti C. (2019). Python serial port access library. Contribute to pyserial/pyserial development by creating an account on GitHub. Available online at: https://github.com/pyserial/pyserial

[B5] Peirce J. W. (2009). Generating stimuli for neuroscience using PsychoPy. Front. Neuroinform. 2:10. 10.3389/neuro.11.010.2008 19198666 PMC2636899

[B6] Straw A. D. (2008). Vision Egg: an open-source library for realtime visual stimulus generation. Front. Neuroinform. 2:4. 10.3389/neuro.11.004.2008 19050754 PMC2584775

